# Correlation of Bethesda Categories in Thyroid Lesions With Histopathology: A Single Tertiary Care Center Experience

**DOI:** 10.7759/cureus.94529

**Published:** 2025-10-14

**Authors:** Sreenidhi Sreeram, Leena Dennis Joseph, Subalakshmi Balasubramanian

**Affiliations:** 1 Pathology, Sri Ramachandra Institute of Higher Education and Research, Chennai, IND

**Keywords:** histopathological correlation, the bethesda system for reporting thyroid cytopathology (tbsrtc), thyroid, thyroid cytology, thyroid fnac

## Abstract

Introduction

Thyroid nodules can be identified primarily through physical examination and history; however, there is no single test to evaluate a thyroid nodule, especially to assess the risk of malignancy (RoM). Hence, a triple assessment through clinical examination, radiological imaging with Thyroid Imaging Reporting and Data System (TIRADS) scoring, and fine needle aspiration cytology (FNAC) is done. The 2023 Bethesda System of Reporting Thyroid Cytology (TBSRTC) is used for the risk stratification of thyroid lesions by FNAC, which will help in management decisions.

Materials and methods

This is a retrospective cross-sectional study conducted at a tertiary care center in Chennai to compare and correlate 101 thyroid histopathology cases with their corresponding cytology smears over one year. The FNAC smears were categorized into one of the six categories according to TBSRTC. The performance of the thyroid cytology was assessed by calculating parameters such as specificity, sensitivity, and RoMs. The results of the study were then compared with the established RoM in TBSRTC and published literature.

Results

Among the 101 cases, 65 (64.35%) were benign and 36 (35.64%) were malignant. Papillary thyroid carcinoma (PTC) was the most common type of malignant tumor with 30 cases (83% of malignancies). Out of the six Bethesda categories, Bethesda category II (benign) had the least RoM (18%), but in comparison with the established rates of TBSRTC and other published studies, it was observed to have a higher RoM. Category III (atypia of unknown significance) has 12 cases, of which three were malignant with a RoM of 25%. Category IV (follicular neoplasm) had the most discordance from histopathology and had a higher RoM (50%) than the established TBSRTC rates. The sensitivity of the current study was 68.57%.

Conclusion

The current study reveals higher RoMs than published studies. Maximum concordance between histology and cytology can be achieved by avoiding aggressive aspiration techniques, a few amendments to reporting criteria, making them less subjective, and periodic quality assurance procedures to improve the accuracy of diagnosis. Moreover, assistive and adjunct methods like immunocytochemistry and molecular testing can also be used in indeterminate lesions.

## Introduction

India has 42 million people who have been estimated to have a thyroid disease encompassing hypothyroidism, hyperthyroidism, autoimmune thyroid disease, and thyroid nodules [1]. Approximately 8.5% of the population presents with a thyroid nodule. Among the Indian population, the most common age group to be affected by a thyroid nodule was 31-40 years of age [2]. 

Thyroid nodules can be multinodular goiter, colloid nodules, inflammatory thyroid lesions, or cancer. Females are more commonly affected than males. Thyroid cancer has less than 5% incidence rate [3]. Benign thyroid nodules rarely cause any symptoms other than swelling, like dysphagia and dyspnea. Sudden change in size, sudden pain, and hoarseness of voice are to be observed as these are signs of aggressiveness of the thyroid nodule. These nodules are usually evaluated by a triple assessment that includes clinical examination (inspection, palpation), radiological imaging, and fine needle aspiration cytology (FNAC) or biopsy.

FNAC of the thyroid is a cost-effective and minimally invasive modality of investigation to identify the nature of the nodule, especially in correlation with radiological imaging of the gland. The Bethesda System of Reporting Thyroid Cytology (TBSRTC) and Thyroid Imaging Reporting and Data System (TIRADS) together aid in patient management according to the risk of malignancy (RoM). 

TBSRTC was first introduced in 2010, was modified in 2017, and was recently updated in 2023. The amendments made in the most recent update mainly include unifying categories to belong under a single term, refining RoM based on more extensive studies, subcategorization of AUS, harmonizing with World Health Organization (WHO) terms, and broadening the follicular cell-derived thyroid carcinoma chapter, among a few other changes [4].

TBSRTC has six categories, which are assigned to the cases based on the presence of certain cytological characteristics. Category I is non-diagnostic, assigned when the smear does not meet the 60-cell adequacy criteria. Category II is benign, with benign follicular cells and no papillary nuclear features or atypia. Category III is atypia of unknown significance, an intermediate category with lesions having features that cannot be deemed suspicious enough to be put in category IV, V, or VI. In the older editions of Bethesda, category III included follicular lesion of unknown significance (FLUS), which has been removed in this edition. Category IV is follicular neoplasm, with a characteristic microfollicular pattern. Categories V and VI are suspicious for malignancy and positive for malignancy, respectively, assigned when there are nuclear features of papillary thyroid carcinoma (PTC) or cytological features of other malignancies.

The management of these patients usually includes follow-up or surgical excision of the nodule by a hemi- or total thyroidectomy with or without a radical neck dissection based on the diagnosis and presence of nodal involvement. The results from thyroid FNACs are generally reported using the various TBSRTC categories. Each Bethesda category has its own established risk of malignancy calculated by considering large studies done worldwide.

This present study aims to compare and assess the diagnostic concordance between the FNAC and histopathology of the thyroid lesions in our institute, to calculate the sensitivity, specificity, and positive predictive values of different TBSRTC categories, and to compare our results with the RoMs of the established, updated TBSRTC categories and published studies.

## Materials and methods

This is a retrospective cross-sectional study conducted at Sri Ramachandra Medical College and Hospital in Chennai for over a year. It includes all hemi- and total thyroidectomy cases that had a prior FNAC done. All thyroidectomy cases that did not have prior FNAC and those that did not have all the necessary information and data were excluded from the study. Ethical approval was obtained prior to the data collection process from the Institutional Research Ethics Committee of Sri Ramachandra Institute of Higher Education and Research (approval number: CSP-MED/25/JUN/117/141).

Selected hematoxylin and eosin (H&E)-stained sections from thyroidectomy specimens of 101 patients were examined. The corresponding FNAC smears were retrieved, examined, and categorized according to TBSRTC. In our institute, FNACs are done with and without image guidance. Guided FNACs are performed by trained interventional radiologists, whereas non-guided FNACs are done by both trained pathologists and pathologists in training under proper guidance. The smears are divided into two sets: one is air-dried and the other is alcohol-fixed. H&E and May-Grunwald-Giemsa staining are followed for the alcohol-fixed and air-dried slides, respectively.

Adequacy of the smears is assessed as part of rapid on-site evaluation (ROSE) by following the rule of 60 cells (six groups of 10 cells each) provided by TBSRTC. In case of inadequacy by ROSE, another pass is made by the pathologist, or it is informed to the radiologist responsible, for reaspiration. The cytology smears from the thyroid are reported and placed under one of the six TBSRTC categories.

The histopathology and corresponding prior cytology smears were compared and correlated. The concordance and discordance were sought. The specificity, sensitivity, and RoMs were calculated. Clinicopathological characteristics such as age, sex, and TIRADS score, if available, were retrieved from the institute's laboratory information system and were stored in a password-protected computer to maintain the confidentiality of the patients.

Parameters like sensitivity, specificity, accuracy, and predictive values (positive and negative) of the thyroid FNAC were assessed. The results of category I lesions were excluded from statistical analysis of the rest of the categories so as to give a more accurate result and for ease of analysis.

## Results

In this study, 101 thyroidectomy cases were reviewed, out of which 65 (64.35%) were diagnosed as benign and 36 (35.64%) were diagnosed as malignant. Seventy-seven (76.23%) were female and 24 (23.76%) were male patients. The youngest patient was 18 years old, and the oldest was 80. The average age at diagnosis of malignancy was 48.58 and of benign was 46.58. Among the 36 cases that were malignant, the most common type was PTC with 30 cases (83% of malignant cases), followed by poorly differentiated carcinoma (three, 8.3%), follicular thyroid carcinoma (two, 5.5%), and medullary thyroid carcinoma (one, 2.7%). Three out of the 30 PTCs were diagnosed as papillary microcarcinoma (<1 cm in size).

The distribution of cases observed among the six categories of TBSRTC was as follows: Category I, which is non-diagnostic (ND), had one case (1.2%), which was a female patient. Most of the female patients (48, 62.4%) were diagnosed as Bethesda category II. Among the female patients, Bethesda categories III, IV, and V had nine (11.7%), 10 (13%), and five (6.5%), respectively. Only four females (5.2%) had Bethesda category VI lesions. Fifty percent (12) of the male patients had category II lesions. Three (1.2%) of the males had category III lesions. Categories IV and V had two (8.3%) male patients under each. The second most common (five, 20.8%) diagnosis made among males, after category II, was category VI. The distribution of cases among the sexes is reflected in Table 1.

**Table 1:** Distribution of cases under each categories between the sexes

Bethesda category [3]	Number of females (N=77)	Number of males (N=24)	Total number of cases (N=101)
I (non-diagnostic)	1 (1.2%)	0 (0%)	1
II (benign)	48 (62.4%)	12 (50%)	60
III (atypia of undetermined significance)	9 (11.7%)	3 (12.5%)	12
IV (follicular neoplasm)	10 (13%)	2 (8.3%)	12
V (suspicious for malignancy)	5 (6.5%)	2 (8.3%)	7
VI (positive for malignancy)	4 (5.2%)	5 (20.8%)	9

The only category I lesion turned out to be a thyroid follicular nodular disease by histopathology. This case is excluded from the statistical analysis of the rest of the five categories for ease of analysis and a more accurate result, as the total number of cases (N) becomes 100.

The majority (60, 60%) of the cases were considered category II (benign), out of which 82% (49) of Bethesda II cases were concordant with the corresponding histopathology diagnosis and 18% (11) were diagnosed as malignant by histopathology. The RoM of category II was 18%.

This was followed by categories III (AUS) and IV (follicular neoplasm), which had 12 each, out of 100 total cases. Seventy-five percent (nine cases) of FNACs diagnosed as category III were benign, and 25% (three cases) were malignant by histopathology, making the RoM 25% for category III. Fifty percent (six cases) of the FNACs with category IV were benign, and 50% (six cases) were malignant by histopathology. Hence, the RoM of category IV became 50%. All the category V (seven cases, 100%) and VI (nine cases, 100%) lesions were malignant by histopathology with a 100% RoM. The RoM of various categories after cytohistopathologic correlation is tabulated in Table 2. Table 3 shows the 2×2 confusion table, and Table 4 shows the accuracy parameters of FNAC by the Bethesda system.

**Table 2:** Risk of malignancy of each Bethesda category in the current study by correlating with histopathological diagnosis Risk of malignancy (RoM) is expressed in percentage. Categorical outcomes were compared between study groups using the chi-squared test. A p-value of <0.05 was considered statistically significant.

Bethesda category [3]	Total number of cases within category	Histopathology diagnosis		RoM	Chi value	P-value
		Number of benign cases (N)	Number of malignant cases (N)			
		Percentage within category (%)	Percentage within category (%)			
I	1	1	0	0	18.448	<0.001
		100%	0%			
II	60	49	11	18%		
		82%	18%			
III	12	9	3	25%		
		75%	25%			
IV	12	6	6	50%		
		50%	50%			
V	7	0	7	100%		
		0%	100%			
VI	9	0	9	100%		
		100%	100%			

**Table 3:** 2×2 confusion matrix *In FNAC diagnosis, positive includes Bethesda [3] categories III, IV, V, and VI, and negative includes category II. FNAC: fine needle aspiration cytology

FNAC diagnosis	Histopathological diagnosis	
	Malignant (N=36)	Benign (N=64)
Positive*	25 (69.4%)	15 (23.4%)
Negative*	11 (30.6%)	49 (76.56%)

**Table 4:** Predictive validity of the thyroid FNAC diagnosis by the Bethesda System of Reporting Thyroid Cytology FNAC: fine needle aspiration cytology

Statistical parameter	Value	95% CI
Sensitivity	69.44%	51.89% to 83.65%
Specificity	76.56%	64.31% to 86.25%
Positive likelihood ratio	2.96	1.81 to 4.85
Negative likelihood ratio	0.4	0.24 to 0.67
Positive predictive value	62.5%	50.45% to 73.18%
Negative predictive value	81.67%	72.77% to 88.13%
Accuracy	74%	64.27% to 82.26%

## Discussion

FNAC is an imperative pre-operative investigative modality that decides the management and follow-up of patients, making the accuracy of the FNAC report of paramount importance. As there is no universal protocol for FNAC procedure or processing techniques, there should be enough quality assurance checkpoints at the institutes/laboratories for accurate and precise reporting [5].

Poor cellular yield in category I smears (one, 0.9%) can be due to incorrect aspiration techniques, like aspirations that take an excess amount of time, using wide-bored needles, and repetitive passes around the lesion, leading to hemorrhagic samples [6,7]. Prolonged stay of the sample within the admixed blood can also lead to clot formation within the hub. The other reasons for non-diagnostic samples can be faulty smear preparation, as well as the cystic nature of the lesion, which yields suboptimal cellularity that does not meet the 60-cell criteria proposed by TBSRTC. The low number of category I samples in the present study is due to the revised ways of proceeding with unsatisfactory samples by communicating with the radiologist or cytopathologist doing the procedure, to repeat the procedure immediately or on a later date, if it is hemorrhagic, for the hemorrhage to settle.

Category II has a higher false negative rate (18%) in comparison with the overall existing Bethesda category. Three of the cases that were malignant by histopathology were lesions of size <1 cm, microscopically measured. Many of the smears were from blind FNAs without imaging guidance, which could greatly impact the diagnosis since the diagnostic lesion cannot be properly visualized. Three other cases had overlapping nuclear features of lymphocytic thyroiditis and PTC, which resulted in discordance between cytology and histopathology. Two cases, despite good cellularity, had artifacts like blood clots and fibrin material that were obscuring the cellular morphology, leading to the false negative report. Abou-Foul et al. emphasized that most of the false negative cases in categories I and II were associated with the small size of the lesion, mostly less than 1 cm, hindering the sampling. The RoM can be significantly influenced by selection bias in categories I and II, since the surgical decision is made on clinical and radiological grounds and might mostly end up not being excised. Some studies have shown that barely 10% of cytologically benign cases had surgery, complicating the establishment of valid accuracy rates [5,8-10].

Category III (AUS) is the most ambiguous class, which includes the smears that did not fit under any of the other categories, especially when the atypia does not reach the level to push the diagnosis into category V. Even then, it is important to raise suspicion as AUS lesions will be followed up with repeat FNAC or thyroidectomy, which could be beneficial for the patient. Since the characteristics are subjective, there is a great level of discordance among many pathologists. Characteristics that raised suspicion among the pathologists in the current study were high cellularity and overlapping nuclear features between oncocytes and PTC features, especially in smears that had significant artifactual hindrance [11]. The majority of false positive cases had background lymphocytic thyroiditis by histopathology, which might have been the reason for the nuclear features. One of the category III cases was diagnosed as non-invasive follicular thyroid neoplasm with papillary-like nuclear features (NIFTP) by histopathology, which, although it was not malignant, had the nuclear features resulting in the AUS diagnosis. Correlating these features with the TIRADS score of the same lesions, category III was assigned. The current study had a comparable RoM of 25% with that of the published Bethesda RoM (22%).

The category with the most discordance in this study was category IV, as seven out of 12 (58%) cases were not diagnosed as follicular adenoma or carcinoma by histopathology. They were instead diagnosed as one of the following: follicular nodular disease (three, 25%), PTC (two, 16%), or poorly differentiated thyroid carcinoma (two, 16%). One (8.3%) of the cases was diagnosed as a follicular subtype of PTC, which can be attributed to the overlapping follicular pattern in cytology smears, leading to categorization into category IV. Adenomatoid nodules are known to have closely packed microfollicles, which may be attributed as one of the overlapping features for falsely diagnosing a follicular neoplasm in FNA smears. Aspirations from a lesser diagnostic, but cellular, area of the same lesion can result in cellular smears with vague microfollicles and a lack of colloid. Since there is no specific or stringent cut-off for the number of microfollicles to be seen in a smear to consider a follicular neoplasm, there is a high inter-observer variability, which may be a major contributing factor for the discordance [12,13]. The diagnosis of category III and IV lesions through FNAC is explored in various studies from other nations, including Korea, in which the diagnosis of category IV was 10% by cytology and most cases that were histologically follicular adenoma/carcinoma were diagnosed as category II or category V/VI (PTC) by cytology [14,15]. In the study by Deandrea et al., the smears diagnosed as follicular lesions of indeterminate significance and follicular neoplasm had a RoM of 4.95% and 25% each. Our study had a higher RoM than the abovementioned studies in category IV [16].

Good accuracy was observed in categories V and VI, having 100% RoM and high true positivity rates. Inter-observer agreement by consensus of cytopathologists with expertise in thyroid cytology resulted in low false positivity rates. Along with it, the correlation of the features with radiological TIRADS score and clinical presentation is of significant importance to give a confident Bethesda category. Comparison of RoM of the current study with two other studies is given in Table 5.

**Table 5:** Comparison of risk of malignancy of the current study with the published Bethesda values and another study Risk of malignancy (RoM) is expressed in percentage.

Diagnostic category	RoM %		
	Bethesda [3]	Alaus et al. [7]	Current study
Non-diagnostic	13%	20%	0%
Benign	4%	14.3%	18%
Atypia of undetermined significance	22%	18.8%	25%
Follicular neoplasm	30%	26.1%	50%
Suspicious for malignancy	74%	84%	100%
Malignant	97%	96.8%	100%

Use of correct aspiration techniques that will yield good smears and evaluation for cellular adequacy by ROSE is important for an accurate diagnosis. Image guidance for FNA, which has become a norm in recent years, has been an essential tool aiding in the correct localization of nodules and choosing the nodules of interest. But not all radiologists are sensitized about the shortcomings of certain aspects of an FNA procedure that experienced cytopathologists avoid. Using a fine-bore needle, avoiding prolonged aspiration duration, doing a residual pass after aspirating the fluid in cystic lesions, smearing immediately after aspiration, and avoiding double smearing are things to be noted by resident cytopathologists and resident radiologists to get a good-quality FNA smear [17].

Appropriate use of both TIRADS and the Bethesda system is important for the accurate diagnosis and categorization of thyroid lesions, which aids in proper clinical decision-making and surgical management. However, there is a need for universally acceptable protocols to prevent a less subjective approach [18]. Table 6 shows the recommended management of each category.

**Table 6:** Recommended management by Bethesda system for each category FNA: fine needle aspiration

Diagnostic category	Usual management
Non-diagnostic	Repeat FNA with ultrasound guidance
Benign	Clinical and sonographic follow-up
Atypia of undetermined significance	Repeat FNA, molecular testing, diagnostic lobectomy, or surveillance
Follicular neoplasm	Molecular testing, diagnostic lobectomy
Suspicious for malignancy	Molecular testing, lobectomy, or near-total thyroidectomy
Malignant	Lobectomy or near-total thyroidectomy

Adjunct immunocytochemistry (ICC) in cell block, liquid-based cytology, and cell transfer methods, even though it is more difficult to examine than immunohistochemistry, is useful in indeterminate and non-diagnostic samples. This can be especially useful in category III lesions and lesions with overlapping features. This can reduce reaspiration rates and decrease diagnostic surgeries [19].

Additional molecular testing in the FNA samples has been studied and researched, aiding in the diagnosis, therapy, and overall prognosis. One such study by Rossi et al., aiming at indeterminate lesions, suggests the use of molecular methods to identify markers that are specific to a type of malignancy, like BRAF and RAS. The study also includes the use of mRNA classifiers and miRNA classifiers for the molecular characterization of thyroid lesions by FNA [20].

With recent advances in the use of artificial intelligence (AI) in histopathology and cytology, there have been studies applying AI in thyroid cytology to improve accuracy in diagnosis. Results from a study by Lee et al. showed better accuracy and sensitivity by AI (Inception ResNet v2) in comparison with human expert cytopathologists [21].

Utilization of the abovementioned modalities as an adjunct to morphology, especially using ICC in cell blocks, or molecular testing if available/feasible, in category III lesions and those with overlapping features, can greatly improve diagnostic accuracy and thereby result in better clinical decisions.

Limitations of this study include, as mentioned earlier, the inclusion of FNAC samples that only have an excision done on them, skewing the RoM in benign lesions. A larger sample size in a longer study period with molecular and IHC correlation would yield a more holistic study.

## Conclusions

FNAC remains the most cost-effective and accurate method for identifying thyroid nodules and stratifying patients according to their risk. The current study has shown higher RoMs than published studies, particularly in categories II and III. Apart from sub-centimetric lesions, which might be missed by blind aspirates, improper aspiration techniques can result in faulty smears, leading to the incorrect categorization of lesions. Maximum concordance between histology and cytology can be achieved by a few amendments in and standardization of reporting criteria to make it less subjective, besides improving aspiration techniques. Periodic quality assurance procedures should also be followed to improve the accuracy of the diagnosis. Moreover, assistive and adjunct methods like immunocytochemistry, molecular studies, and AI can also be used to aid in the proper management of patients, especially with indeterminate lesions.
